# Antibiotic Resistance to *Mycobacterium tuberculosis* and Potential Use of Natural and Biological Products as Alternative Anti-Mycobacterial Agents

**DOI:** 10.3390/antibiotics11101431

**Published:** 2022-10-18

**Authors:** Roberto Arrigoni, Andrea Ballini, Skender Topi, Lucrezia Bottalico, Emilio Jirillo, Luigi Santacroce

**Affiliations:** 1 CNR Institute of Biomembranes, Bioenergetics and Molecular Biotechnologies (IBIOM), 70124 Bari, Italy; 2 Department of Precision Medicine, University of Campania “Luigi Vanvitelli”, 80138 Naples, Italy; 3 Department of Clinical Disciplines, School of Technical Medical Sciences, “A. Xhuvani”, 3001 Elbasan, Albania; 4 Interdisciplinary Department of Medicine, Section of Microbiology and Virology, School of Medicine, University of Bari “Aldo Moro”, 70124 Bari, Italy

**Keywords:** antibiotics, antibiotic resistance, immunity, microbiota, multi-drug resistant *Mycobacterium tuberculosis* strains, stringent response

## Abstract

Background: Tuberculosis (TB) is an infectious disease caused by the bacillus *Mycobacterium tuberculosis* (*Mtb*). TB treatment is based on the administration of three major antibiotics: isoniazid, rifampicin, and pyrazinamide. However, multi-drug resistant (MDR) *Mtb* strains are increasing around the world, thus, allowing TB to spread around the world. The stringent response is demonstrated by *Mtb* strains in order to survive under hostile circumstances, even including exposure to antibiotics. The stringent response is mediated by alarmones, which regulate bacterial replication, transcription and translation. Moreover, the *Mtb* cell wall contributes to the mechanism of antibiotic resistance along with efflux pump activation and biofilm formation. Immunity over the course of TB is managed by M1-macrophages and M2-macrophages, which regulate the immune response against *Mtb* infection, with the former exerting inflammatory reactions and the latter promoting an anti-inflammatory profile. T helper 1 cells via secretion of interferon (IFN)-gamma, play a protective role in the course of TB, while T regulatory cells secreting interleukin 10, are anti-inflammatory. Alternative therapeutic options against TB require further discussion. In view of the increasing number of MDR *Mtb* strains, attempts to replace antibiotics with natural and biological products have been object of intensive investigation. Therefore, in this review the anti-*Mtb* effects exerted by probiotics, polyphenols, antimicrobial peptides and IFN-gamma will be discussed. All the above cited compounds are endowed either with direct antibacterial activity or with anti-inflammatory and immunomodulating characteristics.

## 1. Introduction

Tuberculosis (TB) is an infectious disease caused by the bacillus *Mycobacterium tuberculosis* (*Mtb*), and is characterized by an elevated rate of morbidity and mortality [1]. According to the World Health Organization in 2019, newly diagnosed TB cases around the world numbered 7.1 million, with 206,030 confirmed cases of rifampicin-resistant multi-drug resistant (MDR) TB [2]. Furthermore, co-infection with human immunodeficiency virus (HIV) seems to aggravate the pathogenicity of *Mtb* [3].

TB treatment is based on the six month administration of a multi-drug combination of the antibiotics isoniazid (INH), rifampicin (RMP), and pyrazinamide (PZA) [4,5]. This regimen can be extended up to nine months if there is extensive disease (longer treatments are required under specific condition, i.e., for XDR infections). Isoniazid inhibits the synthesis of mycolic acids, which represent major components of the mycobacterial cell wall [6]. Rifampicin hampers RNA synthesis, while pyrazinamide alters both the plasma membrane and bacterial metabolism [7,8]. In the first two months of treatment, the above drugs are administered with ethambutol. This, in turn, inhibits arabinosyl transferase, which participates in the synthesis of the mycobacterial cell wall arabinogalactan. Despite the activity of these antibiotics against replicant mycobacteria, the coexistence of dormant bacteria affects the efficiency of the treatment. Thus, a prolonged drug administration is implied, which may lead to the development of MDR strains [9]. In fact, under hostile circumstances, *Mtb* demonstrates the so-called stringent response for surviving [10]. This type of response is mediated by alarmones, which regulate fundamental bacterial processes, such as replication, transcription, and translation [11,12]. The stringent response is mostly based on the downregulation of rRNA and ribosomal protein synthesis, thus, leading to the up-regulation of amino acid biosynthetic operons, which supply amino acids for *Mtb* survival [13,14,15]. Furthermore, the *Mtb* cell wall becomes an insurmountable barrier against antibiotic penetration, using the activation of efflux pumps and biofilm formation [16,17,18].

Even when *Mtb* evokes a robust immune response in the lungs, it can escape from it. In fact, *Mtb* enters alveolar macrophages via receptor-mediated phagocytosis; for its survival to occur, it prevents the formation of the phagolysosome, neutralizing the process of acidification, then, it escapes from lysosomal hydrolases [19,20]. In this context, granuloma also present a habitat where *Mtb* can survive, modulating the host immune response [21]. For instance, in TB patients the release of tumor necrosis factor (TNF)-alpha, an antimycobacterial cytokine, is reduced, which contributes to *Mtb* survival [22,23]. The above data are corroborated by the evidence that in non-tuberculous mycobacteria patients, a defective production of certain cytokines, such as interleukin (IL)-12 and interferon (IFN)-gamma has been detected, thus, abrogating the adaptive immune response against mycobacterial disease [24].

Antimicrobial resistance of *Mtb* poses a serious problem to public health, and, therefore, alternative treatments are under investigation. In the present review, we will place emphasis on natural and biological products as novel remedies to treat TB. Among natural products, probiotics are defined as “live microorganisms which when administered in adequate amounts confer a health benefit on the host” [25]. They exert protective functions, modulating the local immune response, avoiding the phenomenon of bacterial translocation and, in particular, inhibiting the growth of *Mtb* strains [26]. Polyphenols are another class of natural products present in fruits, vegetables, cereals, red wine, and extra virgin olive oil (EVOO) [27]. They possess antioxidant and anti-inflammatory activities and have been used to prevent and/or treat various pathologies [28,29]. Among biological molecules, antimicrobial peptides (AMPs), components of the innate immune system, are endowed with antimicrobial and immunomodulating activities [29,30]. IFN-gamma is a cellular product released by a variety of immune cells (macrophages and T lymphocytes) that exhibits the microbicidal activity of macrophages against *Mtb* [31].

On these bases, specific aims of this review will be the description of the mechanisms of antibiotic resistance against *Mtb*, and the ability of this bacterium to survive in a hostile environment. Finally, novel therapeutic approaches with natural products and biotherapeutics will be illustrated.

## 2. Genetic Factors That Predispose to TB Risk

Genome-wide association studies have identified certain loci that predispose patients to TB risk, such as a single nucleotide polymorphism on chromosome 18q11.2, 14q24.3, and 20p13 [32,33]. Conversely, protective loci have been identified on chromosomes 11p13 and 10q26.2 [34,35,36]. Polymorphisms in the region of class II leukocyte antigens, including rs557011 [T] and rs92713 [G], are associated with increased TB risk [37]. Furthermore, polymorphisms of the vitamin D receptor are associated with TB risk, especially in HIV-negative or Asian patients [38]. Immunologically, mutations in genes encoding IL12p40, IL12Rbeta1, IFN-gammaR1, and IFN-gammaR2 abrogate the IL-12/IFN-gamma axis that is very protective against TB risk [39].

## 3. The Impact of *Mtb* on the Immune System

Most of the research on this specific topic has been conducted in rodent models of tuberculosis, owing to the scarce access to lung tissues of TB patients. Therefore, data illustrated in this section are mostly based on experimental models. From an antigenic point of view, the cell wall of *Mtb* consists of mycolic acid, that surrounds the peptidoglycan layer [40]. Fatty acid synthases (FAs) govern the synthesis of mycolic acids that render *Mtb* resistant to the majority of antibiotics. Therefore, the FAs-II system represents the main target of anti-TB drugs [41]. The *Mtb* cell wall first interacts with macrophages, binding the toll-like receptor (TLR)-2 via ERK and p38 pathways. Then, activated macrophages undergo epigenetic modifications of their genes, i.e., DNA methylation [42]. In this respect, *Mtb*-mediated methylation of the inflammasome NLRP3 leads to its activation [43], while hypermethylation of IL-6 receptor (R), IL-4R, and IL-17R suppresses the same receptors [44]. Notably, mycobacterial-encoded methyl transferases are responsible for modifications of methylation patterns during TB infection with Rv1988 and Rv2966c as the major enzymes involved [45]. Particularly, extensively drug resistant *Mtb* strains (XDR) account for increased methylation patterns of macrophage inflammatory genes, thus, supporting the survival of virulent *Mtb* [46]. Metabolically, *Mtb* binding to TLR-2 activates the AKT-mTOR pathway with the prevalence of the inflammatory subset of macrophages, M1, that secrete IL-12, IL-1-beta and TNF-alpha [47]. Such an initial inflammatory response is essential for the progression of TB infection. Furthermore, in the course of sustained infection, *Mtb* inhibits glycolysis with conversion of the M1-macrophages to the anti-inflammatory subset, M2-macrophages [48]. Then, a decrease of IL-1-beta and increase of IL-10 (an anti-inflammatory cytokine) lead to the suppression of bacterial killing [49]. Another tolerogenic cytokine, transforming growth factor (TGF)-beta, polarizes the immune response towards M2-macrophages and the inhibition of this cytokine may represent a potential target of anti-TB therapy [50]. In this immune scenario, it is worth mentioning that M1 macrophages secrete IL-12, which polarizes the immune response towards the T helper(h)-1 subset. This leads to the subsequent production of IFN-gamma, that, in turn, exerts protective functions against TB infection [51,52,53]. Furthermore, IL-12 stimulation that leads to IL-27 neutralization limits *Mtb* growth, increasing IL-6 and TNF-alpha synthesis [54]. Considered together, all the above data suggests that inflammatory cytokines are microbicidal against *Mtb*, but their exaggerated release may damage the host.

**Figure 1:**
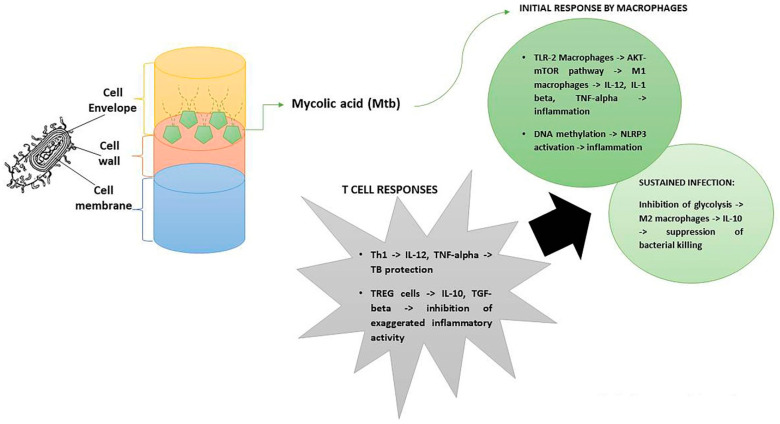
Immune responsiveness during TB infection. Immunity to *Mtb* relies on a fine balance between inflammation (M1 macrophages) and anti-inflammation (M2 macrophages and TREG cells). Cytokines such as IL-12, IL-1-beta, TNF-alpha, and IL-10, according to their cellular source and disease stage, may play both beneficial and detrimental roles.

The role of T regulatory (TREG) cells in the course of TB needs to be clarified. Specifically activated by dendritic cells (DCS) (major antigen-presenting cells) acquire the phenotype CD4+CD25+FOXP3+ [55]. As recently reviewed [56], TREG cells exert different mechanisms of suppression, such as: deprivation of IL-2, which is a growth factor for T cells; secretion of anti-inflammatory cytokines, i.e., IL-10, TGF-beta, and IL-35; granzyme B-dependent killing of target cells; and the inhibition of DC maturation and programmed death-1-mediated suppression of target cells. With special reference to TB, TREG cells exert a functional role, which is dependent on the disease stage. In fact, in the early stage of infection, *Mtb*-mediated activation of TREG cells delays the Th1-mediated protection with the release of IFN-gamma, while in the chronic phase of disease it attenuates the exaggerated inflammatory response [57]. The immune response in the course of TB is illustrated in Figure 1.

## 4. The Gut–Lung Axis

There is consolidated evidence that the gut microbiota is able to modulate both regional and systemic immunity, even including respiratory immune response in the course of TB [58,59]. In this respect, mice deprived of commensal bacteria suffered from severe lung disease but restoration of intestinal microbiota attenuated the disease progression [60,61,62]. Moreover, evidence has been provided that colonization of Helicobacter hepaticus in the murine gut increased susceptibility to TB, along with elevated production of IL-10 [63,64]. Conversely, indole propionic acid, derived from the intestinal commensals, *Clostridia* spp., could reduce the splenic *Mtb* burden in mice [65,66]. The gut–lung axis function is also supported by the notion that antibiotic-mediated depletion of murine gut microbiota increased the *Mtb* load in the lung, promoting the spread of bacilli towards the spleen and the liver [67]. Immunologically, the same mice exhibited more TREG cells and fewer IFN-gamma and TNF-alpha-producing Th1 cells. Conversely, in mice infected with *Mtb*, a depletion of Bacteroidetes and *Clostridia* was observed in the gut [68].

In human recurrent TB, the gut microbiota is enriched in pathogenic bacteria, even including Actinobacteria, Proteobacteria, Faecalibacterium, and Roseburia with a marked reduction of Bacteroidetes and Prevotella [69,70]. In this framework, another important issue is represented by the intestinal production of short chain fatty acids (SCFAs) through the fermentation of dietary fibers. In diabetic patients with increased TB risk, SCFAs were shown to reduce TB-induced release of pro-inflammatory cytokines along with exaggerated liberation of IL-10, thus, promoting TB progression [71].

## 5. *Mtb*-Mediated Resistance against Antibiotics

Despite the availability of antibiotics, TB is increasing in developing and underdeveloped countries. This coincides with the continuous emergence of MDR *Mtb* strains that are resistant not only towards the first-line drugs: isoniazid, rifampicin, ethambutol, and pyrazinamide, but also to fluroquinolones and aminoglycosides, as in the case of extensively drug-resistant TB (XDR TB) and totally-drug resistant TB strains, respectively [72,73]. Resistant strains adopt different strategies to evade antibiotics: i. Increase in cell division under hypotoxic conditions; ii. Utilization of the cell wall as an impermeable barrier against antibiotic access; iii. Efflux pump activation to escape from antibiotics; iv. Biofilm formation [16,17,18,74].

Persistence is a major feature of *Mtb* cells as a few persistent cells can evade antibiotics even at concentrations higher than minimum inhibitory concentrations [75]. Furthermore, persistent *Mtb* cells can sustain the *Mtb* population, maintaining disease status as active. Persistence also implies cellular dormancy, which correlates with increased triacylglycerol and transcription activator protein levels [76,77]. *Mtb* resistance seems to rely on pre-transcriptional mutations and peptidome studies have allowed researchers to better understand the mechanisms of action [78]. *Mtb* adaptive response to antibiotic treatment is mediated by alarmones, that are composed by tetraphosphate guanosine and pentaphosphate guanosine, known as ppGpp [11]. Alarmones allow *Mtb* to survive, modulating biofilm formation, antibiotic resistance, persistence and virulence, demonstrating the so-called stringent response. In Mycobacteria, the alarmones are regulated by the enzyme Rel, which is encoded by the gene rv2583c [79]. In particular, the *Mtb* stringent response relies on the down-regulation of rRNA and ribosomal protein synthesis with the subsequent up-regulation of amino acid biosynthetic operons, supplying amino acids for *Mtb* survival to occur [13,14]. Among major stresses, *Mtb* has to overcome oxidative, nitrosative, and nutrient challenges before infecting macrophages and surviving for years, in the context of a granuloma [80,81]. In addition, PE-PGRS proteins of *Mtb* allow its survival in the granuloma, as well as its interaction with host cells [82,83,84]. Deletion of Rel affects *Mtb* survival or inhibits its growth [10,85]. In fact, the H37Rv delta-Rel-Mtb causes a mild form of lung TB with few granulomas and organ architecture still conserved in comparison to the parental strain, H37rv, which causes a serious pulmonary damage in mice [86]. These data suggest that *Mtb* in the absence of a stringent response cannot induce a chronic status of TB infection. Interestingly, inhibitors of Rel kill *Mtb* and increase antibiotic susceptibility to isoniazid, thus enhancing its microbicidal activity [87].

**Figure 2:**
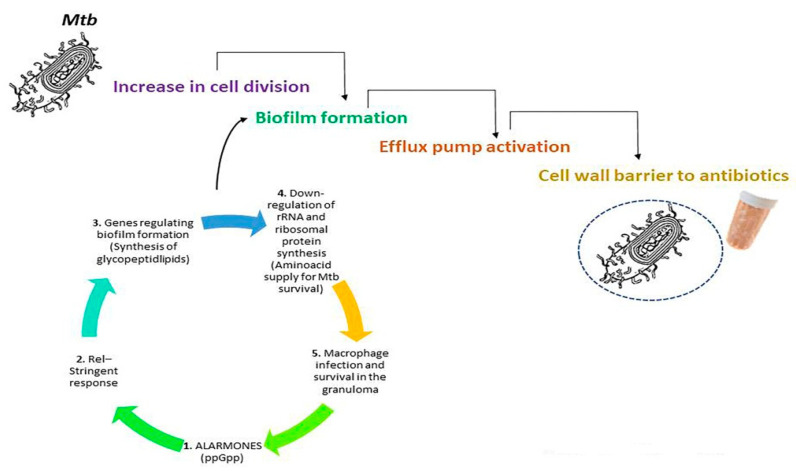
*Mtb*-induced mechanisms of antibiotic resistance. Stringent response elaborated by *Mtb* relies on Rel enzyme through the activity of alarmones. Synthesis of glycopeptidolipids contributes to the formation of biofilms, which increase resistance to antibiotics. Increased supply of amino acids allows *Mtb* to survive for years inside macrophages in the context of granuloma.

Lastly, biofilms with a matrix composed of extracellular DNA, carbohydrates, lipids, and proteins protect *Mtb* from antibiotics [88,89]. In the double knockout deltaRel /deltaRel Z *Mycobacterium smegmatis* strain, evidence has been provided that Rel-induced stringent response regulates the expression of genes involved in glycopeptidolipid synthesis, that are necessary for biofilm formation [90]. Major mechanisms of antibiotic resistance to *Mtb* infection are expressed in Figure 2.

## 6. Novel Treatment to Overcome Antibiotic Resistance against *Mtb*

The emergence of MDR *Mtb* strains has prompted very intensive studies aimed at developing new anti-TB therapies, which are more effective and less toxic. Therefore, in the next paragraphs natural products (probiotics and polyphenols) and biological products (anti-microbial peptides (AMPs) and IFN-gamma), respectively, will be described.

### 6.1. Probiotics

There is evidence that antibiotics against TB alter the gut and pulmonary microbiota [91]. Moreover, antibiotic-mediated dysbiosis may affect the microbiota–immune axis, thus, aggravating the clinical course of TB and increasing the risk of reinfection [65,92]. Probiotics have been identified by the WHO as “Living microorganisms that when administered in adequate amounts as a part of food confer a health benefit to the host” [93]. Just recently, the bactericidal activity of probiotics has been demonstrated to suppress a few antibiotic-resistant superbugs and, therefore, attempts have been made to apply them against tuberculosis [94]. In general terms, probiotics are able to increase the protective effect of the intestinal epithelial barrier, also shifting the immune profile towards a tolerogenic pathway with the production of IL-10 by TREG cells [95,96].

Experimentally, the probiotic Nyaditum resae^®^ (Nr), enriched in heat-killed Mycobacterium manresensis, abrogated the development of active murine TB, through increasing the function of memory TREG cells [97]. Furthermore, Nr effects were evaluated in patients with or without latent TB infection and an increase in T effector cells and memory TREG cells was observed [98]. *Lacticaseibacillus rhamnosus* PMC203, isolated from the vaginal microbiota of healthy women has been shown to exhibit an effective killing of drug-sensitive and drug-resistant *Mtb* infecting RAW macrophages, also inhibiting *Mtb* growth under broth culture medium [99]. *L. casei* supplemented to TB patients for four weeks led to a dramatic reduction of TNF-alpha, IL-6, IL-10, and IL-12, with an up-regulation of metabolites, such as phosphatidylserine, maresin 1, and phosphatidylcholine [100]. *L. crispatus*, isolated from the vaginal microbiota of healthy women, reduced the growth of MtbH37Rv in broth, and MtbH37Rv and *Mtb* XDR TBs in macrophages [101].

### 6.2. Polyphenols

Polyphenols are natural products largely contained in fruits, vegetables, cereals, red wine, and extra virgin olive oil [27]. They are endowed with antioxidant, anti-inflammatory, and microbicidal activities and, therefore, they are currently used in the prevention or treatment of chronic disease [28]. In fact, polyphenols exert their anti-inflammatory activity either inhibiting the NF-kB pathway with reduced expression of pro-inflammatory cytokines or activating TREG cells with the enhanced expression of the anti-inflammatory cytokine IL-10.

**Figure 3:**
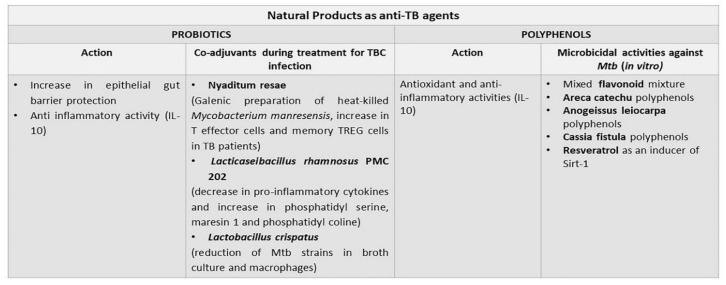
Potential use of natural products as anti-TB agents. Both probiotics and polyphenols have been experimented with as anti-TB pharmaceuticals. Probiotics can modulate the gut–lung axis that is compromised in the course of TB. Polyphenols exert anti-TB microbicidal activity in vitro and their application in human TB needs to be better explored.

To the best of our knowledge, the use of polyphenols in the treatment of TB has been poorly investigated. A mixed flavonoid mixture treatment of TPH-1 infected macrophages and human granuloma reduced the intracellular survival of *Mtb* and increased granuloma formation, with higher levels of IL-12 and IFN-gamma, and lower levels of IL-10 [102]. Polyphenols, extracted from Areca catechu and enriched in catechin, epicatechin, and epigallocatechin, were able to inhibit *Mtb* growth [103]. Polyphenols extracted from the roots of *Anogeissus (A.) leiocarpa* could inhibit Mycobacterium smegmatis growth in view of high concentration of ellagic acid derivatives, ellagitannins, and flavonoids [104]. Clinical data support the use of *A. leiocarpa* in the treatment of cough related to TB. A saponin-polybrophenol antibiotic (CU1), extracted from Cassia fistula inhibited transcription from *Mtb* polymerases, and its ethanol extract was very effective against MDR bacteria [105]. Sirtuin (Sirt) 1 is a NAD-dependent deacetylase, that is able to inhibit apoptosis and inflammation in human cells [106]. Resveratrol, a non-flavonoid polyphenol, is a Sirt 1 activator, which abrogated *Mtb*-induced apoptosis in peritoneal macrophages [107]. Furthermore, Sirt 1 inhibited *Mtb* growth, enhancing GSK3 beta phosphorylation, thus, inducing its deacylation. Therefore, Sirt 1 can be considered as potential anti-TB agent. The use of natural products as anti-TB agents is described in Figure 3.

### 6.3. Antimicrobial Peptides

**Figure 4:**
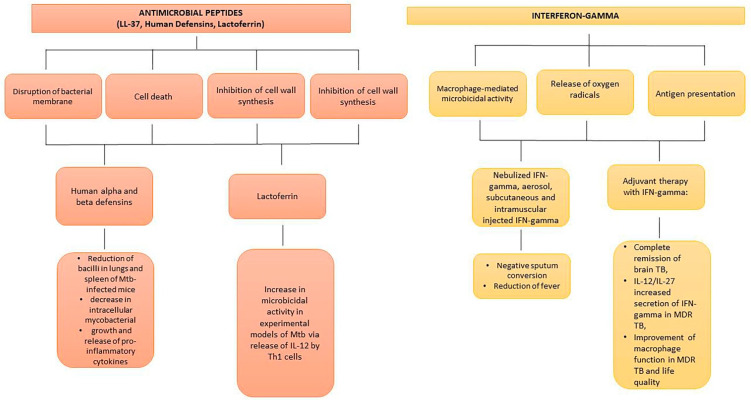
Biotherapeutics for treatment of experimental and human TB. AMPs target *Mtb* in different ways, inhibiting intracellular mycobacterial growth and reducing pro-inflammatory cytokines and IL-12 release by Th1 cells. Nebulized, aerosol, subcutaneous, and intramuscular IFN-gamma administration leads to sputum conversion, reduction of fever, and improvement of life quality in severe human TB.

AMPs have been shown to act as antimicrobial compounds alone or in combination with other drugs [108,109,110,111]. In general terms, they are cationic with less than 50 amino acid residues. Additionally, synthetic analogues are more effective than natural AMPs demonstrating a lower minimum inhibitory concentration [112]. AMPs act through different modalities: i. Disruption of bacterial membrane with formation of pores or via electroporation; ii. Interaction with bacterial components and cell death induction; iii. Inhibition of cell wall biosynthesis; iv. Antimicrobial activity mediated via the activation of the innate immune system [113,114,115]. Cathelicidins, human defensins, and lactoferrin (LF) are the major categories of AMPs. LL-37 is a human cathelicidin, that is able to bind to the negatively charged outer leaflet of the microbial membrane, leading to its disruption [116]. In addition, LL-37 exerts its microbicidal activity against *Mtb* via modulation of the innate immune response [117]. In the course of *Mtb* infection, LL-37 was expressed following the up-regulation of the vitamin D receptor, as well as after oral administration of phenylbutyrate and vitamin D3, with reduction of the intracellular burden of *Mtb* [118,119]. Of note, LL-37 stimulation of infected macrophages led to the production of anti-inflammatory cytokines, IL-10 and TGF-beta, thus, suggesting a modulation of the immune response in the earlier phase of *Mtb* infection [120]. Among analogues of AMPs, acyl depsipeptides (ADEPs) seem to represent a future generation of antibiotics against tuberculosis [121]. ADEPs exert antibacterial activity on *Mtb* strains through activation of the caseinolytic protease ClpP1P2. Biotherapeutics for the treatment of TB are indicated in Figure 4.

### 6.4. Interferon-Gamma and Mesenchymal Stem Cells

IFN-gamma represents a protective cytokine against TB progression that is produced by Th1 and T cytotoxic lymphocytes a few days after infection [122,123,124]. Several genes that encode IFN-gamma and its receptors are required for anti-TB response to occur [125]. In particular, IFN-gamma potentiates the microbicidal response of macrophages, with the release of nitric oxide and reactive oxygen species, and the enhancement of antigen presentation [126]. In TB patients infected with a MDR *Mtb* strain, levels of IFN-gamma were reduced and the same was true in TB patients treated with beta-lactams [127,128]. In a meta-analysis, evidence has been provided that IFN-gamma represents an adjunctive therapy against lung TB [129]. In separate clinical trials, nebulized IFN-gamma, aerosol, subcutaneous injected IFN-gamma, and intramuscularly administered IFN-gamma were very effective in negative sputum conversion and reduction in fever symptoms [130,131]. Remarkably, intramuscular injection of IFN-gamma was very effective in patients with MDR TB [132]. Another three studies in patients with MDR TB demonstrated the efficacy of adjuvant therapy with IFN-gamma: i. A case of brain TB with complete remission following 12 months of treatment [133,134]; ii. Increase in IL-12/IL-27-induced secretion of IFN-gamma in MDR TB [130]; iii. Improvement of macrophage function in MDR TB patients [135]. Finally, adjunct therapy with IFN-gamma was very beneficial in patients with destructive lung TB, also significantly improving life quality [136]. Mesenchymal stem cells (MSCs) are pluripotent stem cells present in the bone marrow, skin, thymus, placenta, and umbilical cord tissue, that can be induced to differentiate into osteoblasts, adipocytes, chondroblasts, and neuron cells [137,138]. Over recent years, a few reports have attributed a role to MSCs in the regulation of tuberculosis granuloma (TG). These cells generate nitric oxide, which can inhibit the growth of *Mtb* within the granuloma [139]. Conversely, MSCs are niches for *Mtb* dormancy in the context of TG and may contribute to the development of Tuberculosis [140]. Notably, there is evidence that MSCs can affect lipid homeostasis in macrophages, also reducing the expression of MHC II [141]. These activities, in turn, may decrease the *Mtb* energy supply, regulating the lipid metabolism in macrophages. As reported by Zhang and associates [141,142], the application of MSCs for the treatment of tuberculosis is at the beginning stages and some issues should be taken into consideration, such as, tissue sources of MSCs, culture conditions in vitro, and possible side effects.

## 7. Conclusions and Future Perspectives

Nowadays, the emergence of MDR *Mtb* strains represent a serious health problem considering the notion that HIV exacerbates human TB pathogenicity. Alternative treatments to anti-TB antibiotics are the objective of current investigations. As reviewed by [143], pharmacomicrobiomics investigates the relationship between drug and gut microbiota with the aim to correct alterations of gut microbiota and treat several diseases, including human lung TB. Polyphenols have scarcely been exploited as anti-TB agents, despite their ability to induce activation of IL-10 [144]. In our opinion, in the later phase of human TB, polyphenols even in conjunction with antibiotics may reduce the exaggerated inflammation, which culminates to destructive lung TB.

Among non-animal AMPs, antibiotics produced by *Lactococcus lactis* exert anti-tubercular activity [145]. Especially, nisin A and lacticin 3147, as well as their mutated variants, are very effective against some MDR *Mtb* strains. With special reference to alarmones, use of photo-cross-linkable (p) ppGpp has allowed researchers to identify specific targets of alarmones [146]. This method seems to be very useful to study the stringent response demonstrated by all mycobacterial species. With special reference to essential oils, evidence has been provided that those extracted from *Micromeria (M.) barbata, Eucalyptus globulus*, and *Juniperus excelsa* in vitro possess antibacterial activity against *Mtb*, even including MDR strains [147]. The reported data are encouraging for in vivo trials mostly with essential oils from *M. barbata.* Despite the availability of natural and synthetic products, more clinical trials are needed to validate their full efficacy as alternative treatment to anti-TB antibiotics in the case of MDR *Mtb* strains.

## Data Availability

All available data are reported in the text.
